# Adaptive learning rate methodologies and clipping mechanisms based upon gradient entropy

**DOI:** 10.1038/s41598-026-60537-3

**Published:** 2026-07-02

**Authors:** Bomin Liu, Jun Yang, Yan Zhu

**Affiliations:** School of Design and Art, Shanghai Dianji University, No.300, Shuihua Road, Pudong New Area, Shanghai, 201306 China

**Keywords:** Scheduler, Optimizer, Learning rate, Entropy-based mechanism, Deep learning, Engineering, Mathematics and computing

## Abstract

Serving as a pivotal parameter during deep learning training processes, learning rate plays a crucial role regarding training efficiency. Traditional optimizers confront challenges such as sluggish convergence and unstable precision; particularly across large-scale datasets, solitary adaptive mechanisms easily lead to premature learning rate decay and training instability, thereby undermining model generalization capabilities. Present research proposes an optimizer enhancement method based upon entropy-modulated mechanisms and multi-strategy integration, improving adaptive capacity of learning rate regulation across diverse training phases through introduction of entropy-aware learning rate scheduling mechanisms while effectively bolstering optimizer responsiveness toward training dynamics and structural complexity. Constructing entropy-modulated optimizers incorporates dual factors of gradient perturbation rate and information entropy to achieve real-time response toward training dynamics, while multi-scale entropy-aware clipping mechanisms are designed to alleviate gradient explosion and structural instability within deep networks. Validated through experiments across multiple public datasets, results demonstrate that present method effectively enhances model robustness and convergence stability.

## Introduction

Optimizer serves as an algorithm in deep learning that adjusts model parameters to minimize loss function, thereby improving model performance. Its main function is to regulate learning rate and update strategy, which together determine training efficiency, convergence speed, and final accuracy. Underlying principle is based on computing gradient of loss function with respect to parameters and updating parameters along gradient direction to reduce error. Traditional stochastic gradient descent (SGD) adopts fixed learning rate for parameter updates, but tends to suffer from slow convergence or entrapment in local optima in complex tasks. To address these issues, adaptive optimizers such as Adam^1^, RAdam^2^, and Lookahead^3^ have emerged, enhancing training efficiency by dynamically adjusting learning rate and incorporating momentum. These adaptive optimizers generally establish update rules based on first- or second-order gradient statistics, yet often lack perception of complexity inherent in training-state information. Such limitation results in instability during non-stationary phases or within models exhibiting frequent structural variations. Accordingly, designing optimization mechanisms with structural awareness of training dynamics has emerged as a promising direction for improving model performance.

To address aforementioned challenges, this study constructs a unified information entropy modeling system that supports optimizer stability and convergence efficiency across diverse task complexities. Specifically, two categories of entropy-modulated schedulers are constructed. Entropy-aware CosineAnnealingLR dynamically adjusts annealing period and minimum learning rate based on entropy variation rate, enhancing adaptability to structural fluctuations. Entropy-aware ReduceLROnPlateau^4^ determines learning rate decay timing through joint assessment of loss variation and entropy stability, while modulating decay magnitude according to current entropy state, thereby enhancing robustness of performance-driven schedulers. Two categories of strategies introduce state-aware mechanisms from perspectives of periodic regulation and performance feedback, respectively, thereby providing enhanced capability for dynamic adjustment during training for optimizers.

In recent years, information entropy has emerged as a valuable mathematical tool for measuring system uncertainty and distributional complexity, and has gradually been introduced into the optimization process of deep learning models^5^. Compared with traditional learning rate mechanisms that rely solely on first-order or second-order statistical characteristics, entropy mechanisms provide supplementary information regarding structural patterns of gradient variation and stability of training states^6^,thereby endowing optimizer design with enhanced perception capability and potential for dynamic regulation. This capability enhances the perception of the optimizer of training dynamics and strengthens its capacity for adaptive regulation. Accordingly, this study further incorporates entropy modulation into two internal aspects of the optimizer, learning rate updating and gradient clipping, to enhance the stability and adaptability of the optimization process.

Specifically, an entropy-aware optimizer is proposed, which incorporates modeling of training perturbations and gradient distributions into the learning rate adjustment process. This design effectively alleviates common issues observed in conventional optimizers during non-stationary training phases, such as annealing failure and premature learning rate decay. This mechanism does not depend on complex parameter combinations and, under current implementation, exhibits strong engineering feasibility and expansion potential^7^. In addition, to enhance the stability of gradient clipping in deep networks, a multi-scale clipping strategy incorporating entropy features is developed. This strategy jointly considers the statistical distribution of gradients and the structural characteristics of the parameter space, enabling hierarchically aware update control^8^. These two mechanisms work together within the learning rate modeling process, ultimately improving the robustness of the optimizer across tasks with varying complexity and data distributions.

The remainder of this paper is structured as follows: Section "Related work" introduces improvements in other adaptive optimizers, learning rate schedulers, and entropy-based methods. Section "Methodology" presents methods proposed in this study for optimizer enhancement, including integration of entropy-based learning rate schedulers and two mechanisms developed based on entropy principles. Section "Experiments and results" describes the experimental setup, including datasets, parameter configurations, and evaluation metrics. Section "Discussion" discusses key elements of this study and compares them with previous related research. Section "Conclusion" provides an analysis of results and a perspective on limitations of this study.

## Related work

### Optimizer

In addition to the widely used RMSProp and SGD, other commonly used adaptive learning rate optimizers include Adagrad, Adadelta, and ranger.

Adagrad^9^ optimizer updates parameters based on two key principles: the accumulation of squared gradients and adaptive learning rates. This design allows for effective control of the learning rate during training and helps to mitigate issues associated with fixed step sizes. However, since Adagrad accumulates all past squared gradients, the effective learning rate can decay rapidly and may approach zero, which can lead to premature termination of training. Adadelta^10^ addresses this issue by removing the need to manually define an initial learning rate. Instead, it uses an EMA^11^ (exponential moving average) Exponential Moving Average of past gradients to regulate the decay process. This increases the complexity of the update rule and may lead to less stable performance.

Ranger21 optimizer integrates eight distinct components to form a synergistic optimizer with unique advantages. Its core components are Adam and Lookahead. Adam is primarily responsible for correcting biases during the training process and stabilizing the convergence of the learning rate. Lookahead reduces update noise by employing parameter moving averages and a dual-weight update mechanism. These two core components effectively address issues such as overly large learning rates in the early stages of training and challenges in adaptive learning rate computation, thereby improving overall optimization performance. However, the Ranger21 optimizer still faces challenges related to long-term decay and the complexity of hyper-parameter tuning within its learning rate mechanisms.

In recent years, beyond adaptive optimizers based on first-order gradient statistics, curvature-aware or approximate second-order optimizers have also attracted widespread attention. Such methods generally construct preconditioning information through Hessian matrices, Gauss–Newton matrices, or corresponding approximations, so as to enhance adaptability of parameter updates to loss landscapes. For instance, Shampoo performs gradient scaling via Kronecker-structured preconditioning matrices, thereby exhibiting strong curvature adaptability in large-scale training^12^; Sophia employs lightweight estimation of diagonal Hessian to construct second-order preconditioning and combines clipping strategies to regulate update magnitude, achieving high efficiency in large-scale model pretraining^13^. In contrast to these approaches, this study does not explicitly estimate Hessian or construct curvature matrices, but instead treats gradient information entropy and perturbation rate as signals for perceiving training dynamics, which are used to regulate learning rate pathways and update control strategies.

### Scheduler

Learning rate schedulers adjust learning rates dynamically throughout training, contributing to accelerated convergence and preventing entrapment in local optima. They are mainly divided into two types: time-based cyclic schedulers and performance-based adaptive schedulers.

CosineAnnealingLR scheduler^14^ decays the learning rate smoothly following a cosine function, maintaining a relatively high value in the early stages of training and gradually reducing it toward stable convergence. It offers advantages such as smooth adjustment behavior and simplicity in parameter tuning. However, its cycle parameter and minimum learning rate typically rely on fixed values, making it difficult to adapt dynamically to changing training conditions. This constraint may lead to reduced flexibility, especially when dealing with complex architectures or non-stationary data distributions.

ReduceLROnPlateau scheduler^15^ triggers learning rate decay by monitoring changes in validation loss metrics, making it suitable for tasks characterized by significant performance fluctuations or abrupt shifts in convergence speed. However, this method relies on a single performance indicator, which makes it vulnerable to interference from local fluctuations. In the absence of structural complexity modeling, it may result in delayed or premature annealing.

Most existing schedulers rely on preset rules or loss changes, lacking real-time perception of structural characteristics in training states, which makes it difficult to accommodate frequently shifting learning stages in deep models. In recent years, some studies have attempted to incorporate uncertainty-based indicators into scheduler design^16^, yet gains in both theoretical foundations and empirical effectiveness remain limited. These challenges underscore a clear rationale and offer significant opportunities for introducing entropy-based mechanisms.

### Entropy modulation mechanism

Traditional optimizers are primarily based on first or second order gradient statistics, which makes it difficult to effectively handle gradient fluctuations and the structural complexity of parameter updates during training, especially during non-stationary phases or in deep neural networks, where problems such as learning rate degradation and training instability are more likely to occur^17^.

Accordingly, this study further proposes an entropy-modulated optimizer, in which two dynamic regulation factors, gradient perturbation rate and gradient information entropy, are introduced on the basis of Adagrad to jointly control parameter update magnitude. Among them, perturbation rate quantifies relative variation of current gradients with respect to previous iteration and serves to identify oscillatory regions during training^18^, whereas information entropy characterizes imbalance degree of gradient distribution and adjusts learning rate from perspective of structural complexity. Existing approaches such as diffGrad^19^and AngularGrad^20^primarily focus on perturbation-based modulation at parameter level, operating on local learning rate adaptation. In contrast, this study models training dynamics from perspective of global gradient variation and integrates entropy mechanisms to enable coordinated control across scheduler and optimizer paths, thereby emphasizing a higher-level form of adaptivity. This design preserves advantages of adaptive learning while effectively preventing premature learning rate degradation and enhancing adaptability of optimization process under complex loss landscapes.

In terms of gradient clipping, traditional methods are mostly based on global thresholds or variance-based adjustments and lack fine-grained control over sensitivity to different hierarchical structures. Therefore, a multi-scale-driven entropy-modulated threshold clipping mechanism is also proposed. This mechanism integrates three types of statistical indicators to construct the clipping threshold, gradient variance, gradient information entropy, and the largest singular value of parameter weights^21^. By combining these three types of information in a weighted manner, the mechanism dynamically generates clipping thresholds that adapt to structural complexity and training states, enabling hierarchical control over the magnitude of parameter updates.

It is worth noting that entropy-SGD^22^represents an early attempt to introduce entropy mechanisms into optimization processes, in which a local entropy regularization term is constructed within a parameter neighborhood to guide gradient descent toward flatter minima, thereby improving model generalization ability. However, this approach essentially alters structure of original loss function and relies on multiple inner-loop sampling steps for optimization. By contrast, gradient entropy mechanism introduced in this study does not modify objective function and instead serves as a dynamic perception indicator during training. By characterizing degree of non-uniformity in gradient distributions, it assists regulation of learning rate trajectories and structural control strategies.

Finally, traditional schedulers lack modeling capability for uncertainty in training states within parameter update rules, making it difficult to adapt to stage-specific fluctuations and non-stationary characteristics during training in deep models^23^. By incorporating information entropy into internal control parameters of schedulers, this study strengthens CosineAnnealingLR and ReduceLROnPlateau in perceiving training states and adapting to structural variations. As a result, entropy-aware schedulers with dynamic adjustment properties are constructed.

## Methodology

### Overall framework

This study constructs an optimizer enhancement framework integrating entropy mechanisms and scheduling strategies to bolster convergence efficiency and training stability during model optimization. Centering upon structural-aware regulation mechanisms driven by gradient entropy, this framework develops two categories of scheduler enhancement modules and two classes of optimizer sub-modules, acting respectively upon learning rate scheduling pathways and gradient update trajectories.

**Fig. 1:**
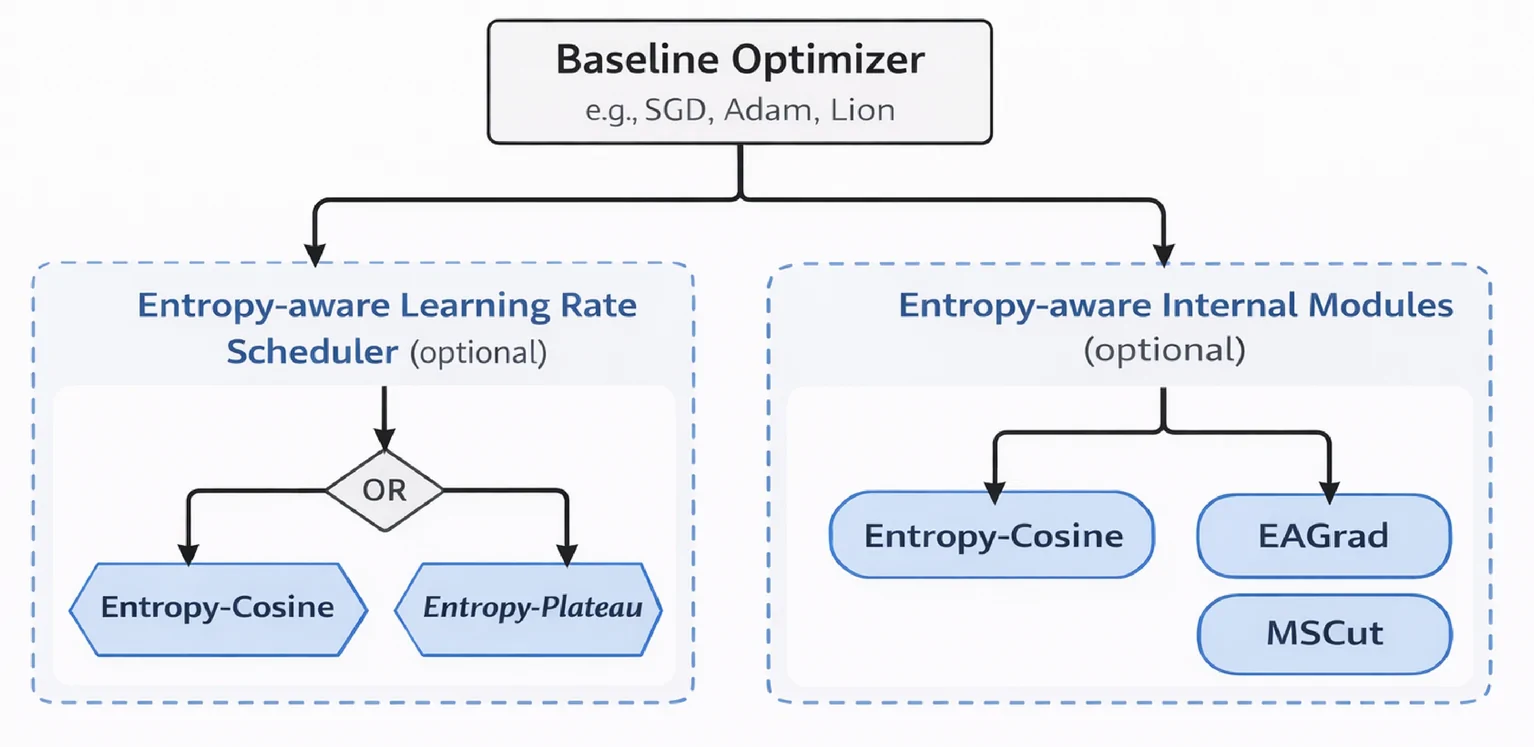
Overall framework architecture.

In the scheduler path, entropy-aware mechanisms are introduced into CosineAnnealingLR and ReduceLROnPlateau, allowing dynamic adjustment of cycle parameters, minimum learning rates, and decay factors, so that learning rate variations can respond to uncertainty in training states. In the optimizer path, an entropy-modulated update mechanism named EAGrad is constructed based on Adagrad, where gradient perturbation rate and local entropy jointly determine the update step size. A multi-scale clipping mechanism, MSCut, is also designed by combining gradient variance, information entropy, and spectral features to achieve stable control in deep neural networks. The following figure illustrates the technical roadmap of this enhancement framework. As illustrated in Fig. 1, entropy-aware schedulers and internal modules are optionally incorporated into a baseline optimizer, where schedulers are mutually exclusive and internal modules act as independent plug-in enhancements.

### Design of entropy-aware scheduler mechanism

To enhance the adaptability and generalization capability of the optimizer at different training stages, this study introduces information entropy as a dynamic indicator of training state, integrating it into core parameter modeling of learning rate schedulers, thereby endowing schedulers with sensitivity to training complexity and uncertainty. Based on existing CosineAnnealingLR and ReduceLROnPlateau scheduling strategies, this study constructs entropy-driven scheduler modules, enabling learning rate updates to move beyond static rules and achieve joint regulation of nonlinearity and task sensitivity guided by entropy signals.

#### Entropy-cosine

CosineAnnealingLR is a scheduler that modulates the learning rate in a cyclical manner based on a cosine function, with its core formulation given as follows:

1 $$\begin{aligned} (lr)_t = \eta _{\text {min}} + \frac{1}{2} \left( \eta _{\text {max}} - \eta _{\text {min}} \right) \left( 1 + \cos \left( \frac{T_{\text {cur}}}{T_{\text {max}}} \pi \right) \right) \end{aligned}$$

where $T_{\text {max}}$ denotes the annealing period; $\eta _{\text {min}}$ and $\eta _{\text {max}}$ correspond to the minimum and maximum learning rates, respectively.$T_{\text {cur}}$ denotes the number of epochs completed within the current training cycle.

To introduce structural perception capability of training states, this study constructs entropy-modulated period parameters and bottom learning rates. The period adjustment is formulated as follows:

2 $$\begin{aligned} T_{\text {max}}^{(t)} = T_0 \cdot (1 + \mu \cdot \Delta H_t) \end{aligned}$$

where $\Delta H_t = |H_t - H_{t-1}|$ denotes the magnitude of information entropy variation between consecutive training epochs, and *μ* represents the sensitivity coefficient for period adjustment. This design enables the period to automatically extend when the training state undergoes substantial changes, thereby preventing frequent oscillations in learning rate from interfering with convergence.

To modulate the bottom learning rate based on training dynamics, its adjustment is defined as follows:

3 $$\begin{aligned} n_{\text {min}}^{(t)} = n_{\text {base}} \cdot (1 - \rho \cdot H_t) \end{aligned}$$

where Higher entropy values $H_t$ indicate that training is in a state of uncertainty, in which case a higher $n_{\text {min}}$ is retained to maintain sufficient exploratory capacity. During periods of entropy decline, the range is automatically reduced to enhance stability. This mechanism implements entropy-aware control from both cycle and amplitude within the annealing function, and enhances responsiveness of learning rate modulation to structural complexity and dynamic convergence stages.

#### Entropy-Plateau

ReduceLROnPlateau determines entry into performance plateaus by monitoring whether validation set performance metrics remain stagnant across multiple consecutive training epochs. Exceeding patience parameters predefined within scheduler triggers a decay operation of learning rate once plateau duration surpasses specified thresholds. Triggering logic adheres to following principles:

4 $$\begin{aligned} {\text {if } loss_t > loss_{t-\text {patience}} \Rightarrow lr_t = lr_{t-1} \cdot \text {factor}} \end{aligned}$$

In actual training, this mechanism may be erroneously triggered by short-term loss oscillations. To improve its stability, this study introduces information entropy as an auxiliary triggering condition, forming an entropy-performance criterion mechanism:

5 $$\begin{aligned} { \text {if} \Delta \,\text {loss}_t< \varepsilon \text { and } \Delta \,H_t < \delta \implies \text {lr}_t = \text {lr}_{t-1} \cdot \text {factor}_t } \end{aligned}$$

where $\Delta \,\textit{loss}_t = |\textit{loss}_t - \textit{loss}_{t-1}|$, $\Delta \,H_t = |H_t - H_{t-1}|$; only when both loss fluctuation and entropy fluctuation remain within a convergent state is learning rate decay permitted, effectively preventing premature annealing during unstable phases. Meanwhile, to enhance adjustment flexibility, the decay factor itself can also be adaptively modulated based on the current entropy value:

6 $$\begin{aligned} { \text {factor}_t = \text {factor}_0 \cdot (1 - \xi \cdot H_t) } \end{aligned}$$

Specifically, when entropy remains elevated, adjustment magnitude is reduced; during stable training phases, a larger decay factor is applied to accelerate convergence. This mechanism substantially improves the robustness of the ReduceLROnPlateau scheduler to training-state fluctuations, enabling more stable and adaptive regulation of learning rates in deep networks throughout prolonged training cycles.

Above formulation adopts a linear decay structure characterized by monotonicity and gradient continuity, which ensures numerical stability while providing sufficient adjustment sensitivity. Consequently, this design circumvents potential gradient saturation issues inherent in nonlinear functions across high-entropy or low-entropy regimes.

Two types of entropy-modulated schedulers proposed in this section respectively enhance structural adaptability of learning rate adjustment from periodic and performance-driven perspectives. These schedulers can operate in synergy with internal parameter update modules within an optimizer.

#### Comparative mathematical analysis of entropy-modulated schedulers

This subsection presents a unified mathematical formulation to compare the differences between the two entropy-modulated schedulers. Let the learning rate update rule be uniformly expressed as follows:

7 $$\begin{aligned} \eta _{t+1} = \eta _t \cdot f(\Delta H_t) \end{aligned}$$

where $\Delta H_t$ represents the entropy variation at the current moment. For entropy-cosine, learning rate update is determined by the magnitude of entropy fluctuation and directly influences the gain coefficient of learning rate:

8 $$\begin{aligned} \textit{f}(\Delta H_t) = (1 + \mu \cdot \Delta H_t) \end{aligned}$$

This implies that entropy variation directly affects the adjustment magnitude of learning rate, enabling smooth modulation of its periodicity, especially during fluctuations between early and late stages of training.

In contrast, learning rate adjustment in Entropy-Plateau scheduler is governed by a joint condition involving entropy variation and loss fluctuation:

9 $$\begin{aligned} \textit{f}(\Delta H_t) = \gamma (H_t) \quad \textit{if} \quad \Delta L_t < \varepsilon \end{aligned}$$

where $\gamma (H_t)$ denotes an entropy-driven decay function, which triggers learning rate reduction only when both loss variation and entropy fluctuation satisfy predefined conditions. In this way, the entropy mechanism primarily governs the timing of decay, preventing premature reduction of learning rate caused by local fluctuations.

Overall, entropy-cosine focuses more on directly adjusting the periodicity of learning rate based on entropy variation, while Entropy-Plateau flexibly controls the timing of learning rate decay by combining loss variation and entropy fluctuation. The choice between the two depends on task complexity and specific performance requirements during training.

### Design of an entropy-modulated dynamically adaptive optimizer

Although Adagrad demonstrates strong robustness in handling sparse gradient scenarios, its update strategy, which relies on the accumulation of squared gradients, tends to cause rapid learning rate decay in the later stages, thereby affecting both convergence efficiency and training stability. To address this issue, a dynamic adjustment mechanism integrating gradient perturbation rate and local information entropy is proposed in this study, leading to the development of an entropy-aware optimizer named EAGrad (entropy-aware Adagrad), based on the Adagrad framework. The goal is to enhance its adaptability in non-stationary gradient conditions. The new learning rate update rule is as follows:

10 $$\begin{aligned} \theta _{t+1} = \theta _t - \frac{n}{\sqrt{G_t + \alpha \cdot \lambda _t + \beta \cdot H_t + \varepsilon }} \cdot \nabla \mathscr {L}_t \end{aligned}$$

where $G_t = \sum _{i=1}^t (\nabla L_i)^2$ denotes accumulated squared gradient sum, *η* represents initial learning rate, and *\varepsilon* refers to constant for numerical stability. Core-improvements are as follows:

11 $$\begin{aligned} \lambda _t = \frac{\left\| \nabla \mathscr {L}_t - \nabla \mathscr {L}_{t-1}\right\| _2}{\left\| \nabla \mathscr {L}_{t-1}\right\| _2 + \delta } \end{aligned}$$

It is defined as gradient perturbation rate, characterizing the degree of fluctuation in gradient updates at the current training stage, where *δ* is a small constant introduced to ensure numerical stability by preventing division by zero,$\mathscr {L}_t$ represents the instantaneous loss value of the model at the *t*-th time step.

Perturbation rate defined in above equation shares formal similarities with methodologies such as diffGrad and AngularGrad, yet exhibits distinct functional disparities. Former serves as internal regulatory factor of optimizer, primarily influencing update pace of individual parameters or directional vectors; conversely, perturbation rate proposed in this paper functions as global training state evaluation signal, constructing training dynamics mapping in conjunction with gradient entropy to drive external scheduling strategies.

12 $$\begin{aligned} H_t = - \sum _i p_i \log (p_i), \quad p_i = \frac{|g_{t,i}|}{\sum _j |g_{t,j}|} \end{aligned}$$

It is defined as the information entropy of the gradient distribution, characterizing the degree of information concentration in the current parameter update.

Adjustment coefficients *α* and *β* regulate the relative influence weights of the perturbation rate and entropy, respectively. This formulation retains the localized adaptivity characteristic of Adagrad, while embedding a real-time responsiveness to training dynamics. As a result, it mitigates stagnation caused by abrupt early-stage gradient decay and enhances long-term optimization performance in complex model training. In subsequent experiments, EAGrad is not employed as an internal module of Ranger21, but is instead treated as an independent optimizer for parallel comparison with entropy-enhanced variants.

**Fig. 2:**
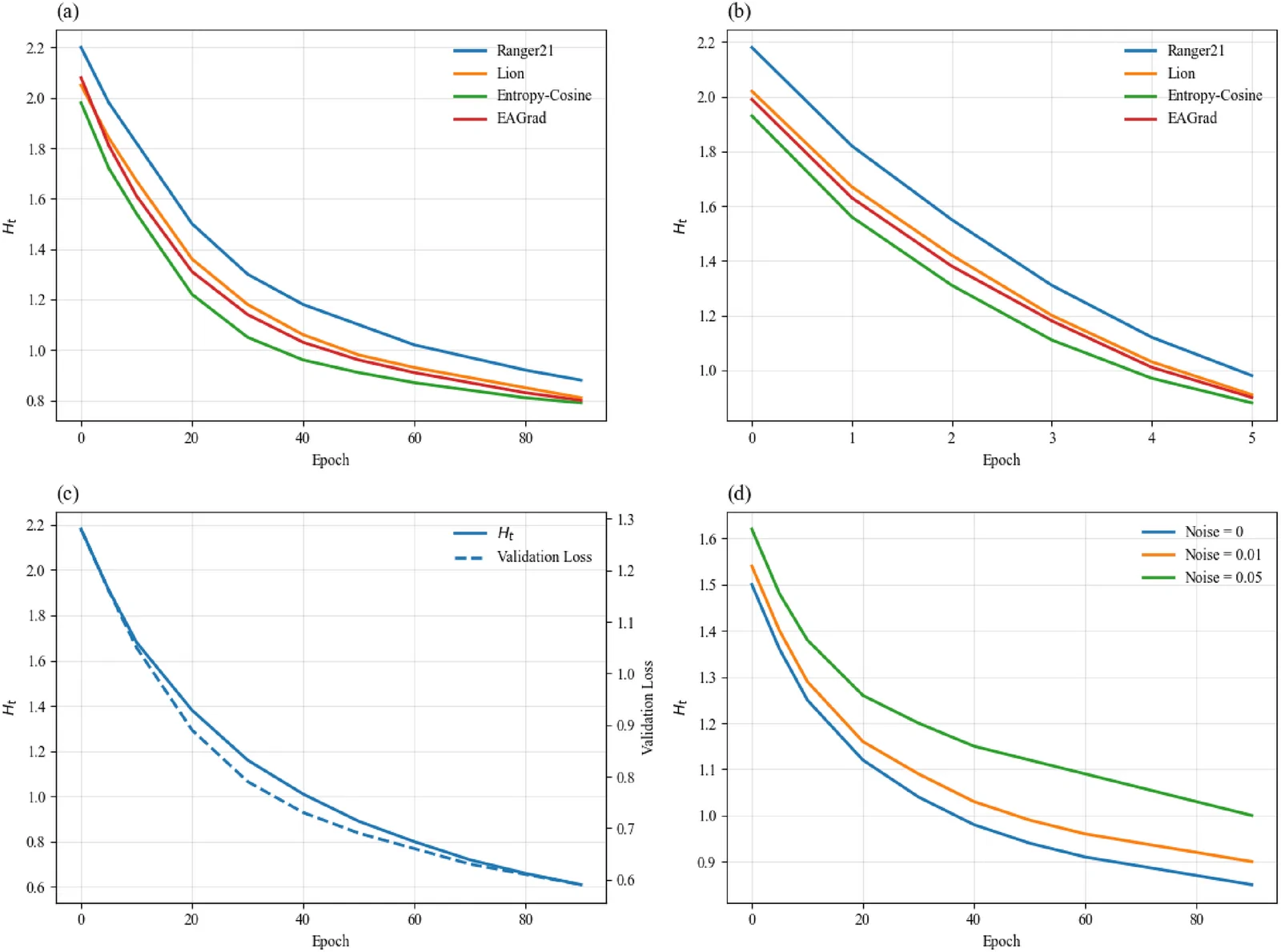
Evolution trajectories of gradient entropy $H_t$ under different tasks, optimizers, and noise conditions, and its relationship with training stability. Among them, (**a**) and (**b**) present trajectories of $H_t$ for different optimizers on CIFAR-10 and SST-2, respectively; (**c**) illustrates corresponding variation between $H_t$ and validation loss on CIFAR-10; (**d**) shows variation of $H_t$ under different gradient noise levels.

Gradient magnitudes across network layers with diverse deep architectures exhibit inherent variance; consequently, this study applies normalization to gradient vectors during calculation of gradient entropy, focusing exclusively on distributional morphology to prevent $H_t$ from being overshadowed by layers dominated by extreme gradient values. Simultaneously, mathematical formulation of entropy depends upon relative distribution rather than absolute gradient magnitudes. Therefore, this metric serves as a characterization index for training dynamics, possessing intrinsic architectural robustness across disparate configurations. To examine variation characteristics of gradient entropy under different training conditions and its relationship with training stability, this study provides comparative visual analysis across tasks and optimization settings, as illustrated in Figure 2.

### Entropy-modulated multi-scale threshold clipping mechanism

In complex deep neural architectures, gradient clipping plays a crucial role in mitigating gradient explosion and enhancing training stability. However, conventional adaptive clipping strategies predominantly rely on simple statistics such as mean and variance to define thresholds, failing to incorporate parameter hierarchy and update complexity effectively. To address this limitation, a multi-scale threshold clipping mechanism named MSCut (multi-scale clipping) is proposed, integrating entropy modulation with tensor spectral analysis to enable more granular and tunable clipping control. Clipping thresholds are defined through the following multi-factor formulation:

13 $$\begin{aligned} \tau _t = \gamma \cdot \sqrt{\textit{Var}(\nabla \mathscr {L}_t)} + \eta \cdot \textit{H}(\nabla \mathscr {L}_t) + \zeta \cdot \sigma _{\textit{max}}(W_t) \end{aligned}$$

where $\textit{Var}(\nabla \mathscr {L}_t)$ denotes the variance of the current gradient tensor, reflecting training volatility.$\textit{H}(\nabla \mathscr {L}_t) = -\sum _i p_i \log (p_i), \, p_i = \frac{|g_i|}{\sum _j |g_j|}$ denotes the local gradient information entropy, quantifying the imbalance in the gradient update distribution.$\sigma _{\textit{max}}(W_t)$ denotes the maximum singular value of the current parameter matrix, characterizing the representational strength and importance of the corresponding network layer, *γ*, *η*, and *ζ* represent the scaling coefficients for the variance term, entropy term, and structural term, respectively. Gradient clipping is performed according to the following correction strategy:

14 $$\begin{aligned} \textit{g}_i' = {\left\{ \begin{array}{ll} \textit{g}_i, & \textit{if} \ |\textit{g}_i| < \tau _t \\ \tau _t \cdot \textit{sign}(\textit{g}_i), & \textit{otherwise} \end{array}\right. } \end{aligned}$$

By jointly leveraging variance, entropy, and singular value metrics, this mechanism enables dynamic discrimination of sensitivity across temporal stages and architectural layers in parameter space. It thereby facilitates a hierarchical and weighted clipping strategy. Compared with conventional approaches, it maintains conservative control during early training phases, while progressively relaxing clipping constraints as the model approaches convergence, achieving both robustness and efficiency. This makes it particularly well suited for deep neural network training in resource-constrained environments.

**Algorithm 1:**
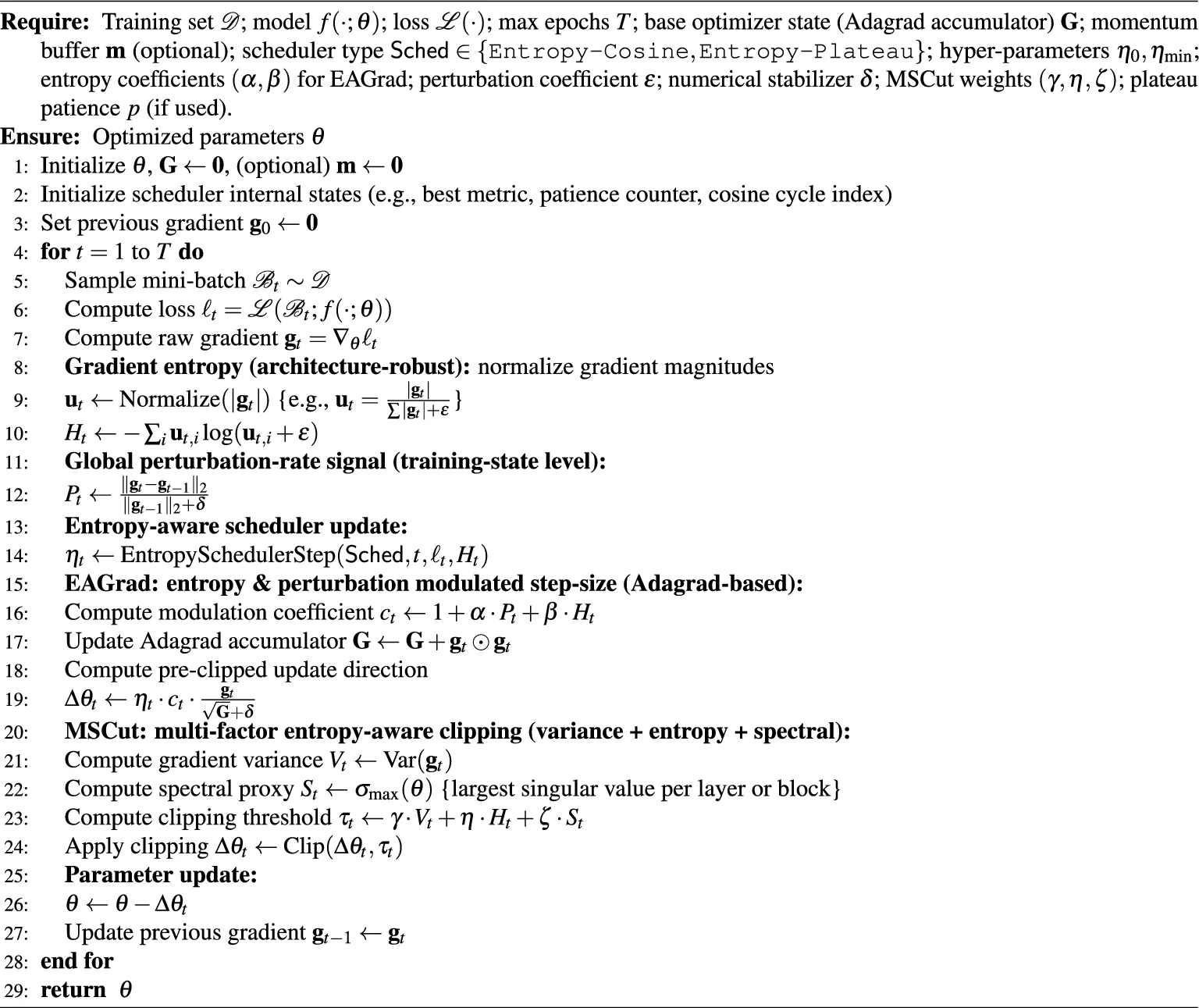
Entropy-mechanism enhanced optimization with entropy-aware scheduler, EAGrad, and MSCut

**Algorithm 2:**
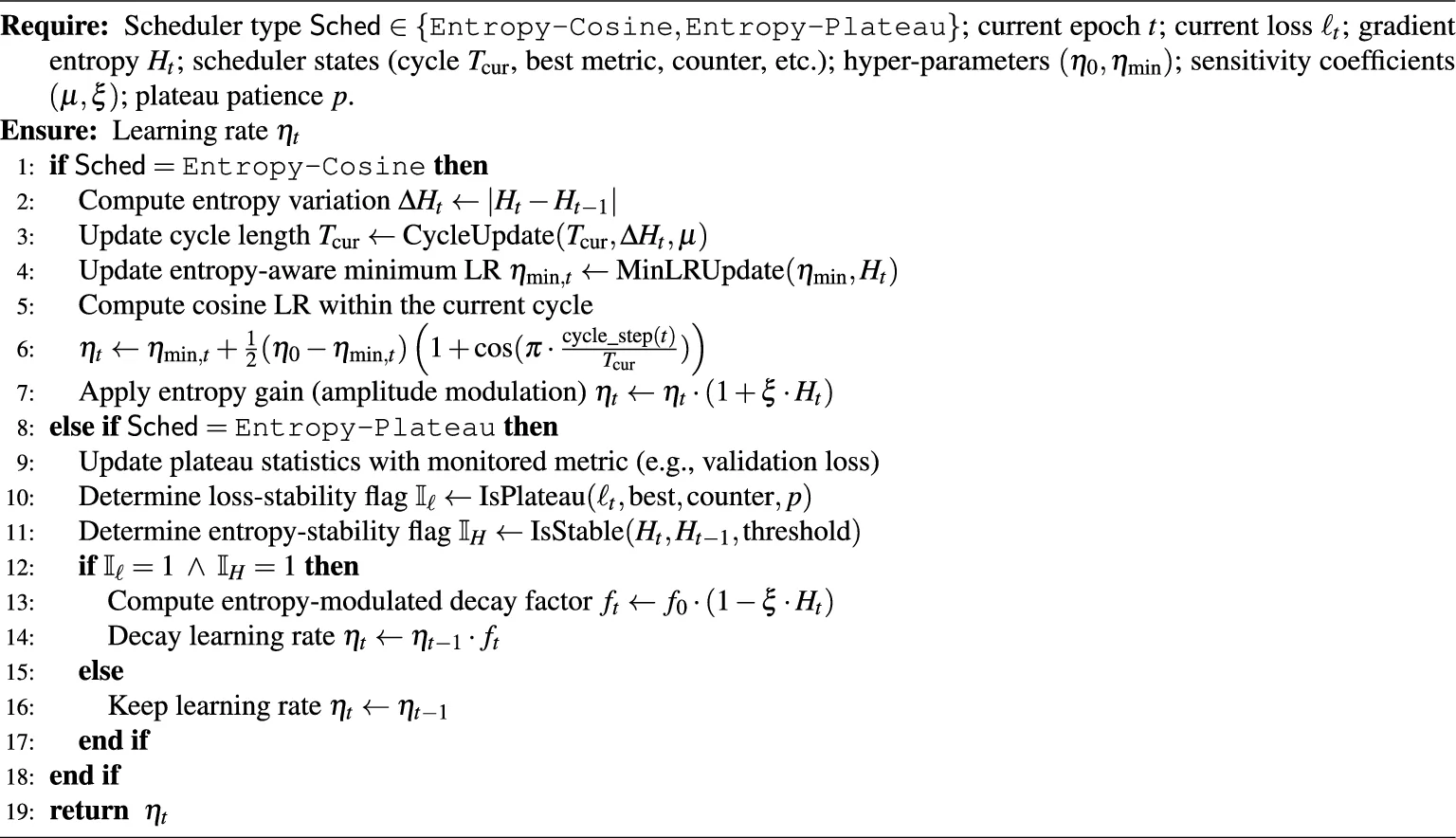
EntropySchedulerStep$$(\textsf{Sched}, t, \ell _t, H_t)$$

## Experiments and results

### Experimental setup

To comprehensively evaluate performance of proposed entropy-mechanism optimizers, experiments encompass two categories of tasks, namely image classification and natural language understanding. Five datasets, CIFAR-10, ImageNet-100, SST-2, ImageNet-1K, and MNLI, are employed, covering medium-scale and higher-complexity training scenarios.

This study categorizes optimizers used in experimental comparisons into three groups: external baseline optimizers Ranger21 and Lion, entropy-enhanced variants constructed upon Ranger21, and independent optimizer EAGrad constructed upon Adagrad. Among them, Entropy-Cosine, Entropy-Plateau, and MSCut are employed to evaluate enhancement effects of entropy mechanisms within Ranger21 framework, whereas EAGrad, as an independent entropy-aware optimizer, is utilized to assess role of gradient perturbation rate and gradient entropy when directly involved in parameter updates. Evaluation metrics include Accuracy as well as training and validation loss, and in selected experiments, convergence speed and training stability are additionally recorded to analyze dynamic adaptability of optimizers across different training stages. For spectral feature term involved in MSCut, experimental implementation adopts approximate estimation of maximum singular value together with a low-frequency update strategy to control additional computational overhead in large-scale model training.

### Analysis regarding optimization behavior on fundamental vision tasks

To analyze optimization behavior of proposed entropy-mechanism methods within typical vision tasks, this section selects base-scale image classification as experimental subject. This task exhibits representativeness regarding data complexity and gradient distribution characteristics, capable of manifesting gradient fluctuations during initial training phases while simultaneously reflecting stability disparities throughout mid-to-late convergence stages; thus, it serves as suitable foundational scenario for comparative analysis across different optimization strategies^24^.

Experiments employ fixed network architectures during training to minimize impact of model capacity variations upon optimization process. All optimizers operate under identical initialization conditions, training epochs, and batch size configurations, with evaluation metrics encompassing final classification accuracy alongside training and validation loss to comprehensively characterize performance regarding convergence efficiency and generalization capability. Among comparative methodologies, Lion^25^-a lightweight optimizer proposed by Meta in 2023-is introduced as a contemporary baseline alongside traditional optimizer Ranger21. Lion demonstrates favorable stability across multiple vision tasks through sign-based momentum updates, thus gaining wide recognition as one of representative optimizers in recent years. Meanwhile, this paper integrates entropy-modulated scheduling and gradient-aware mechanisms into Ranger21 framework to construct diverse entropy-enhanced optimization schemes, serving to verify effectiveness of proposed methods regarding training dynamics perception. Table1 presents overall performance results of different optimizers on this vision task.

**Table 1:** Comparison regarding training and validation performance on fundamental vision tasks.

Optimizer	ACC (%)	T-loss	V-loss
Ranger21	88.3	0.42	0.55
Ranger21-Entropy-Cosine	90.1	0.34	0.43
Ranger21-Entropy-ReduceLROnPlateau	89.8	0.35	0.45
EAGrad	88.8	0.40	0.50
Ranger21-MSCut	89.0	0.39	0.50
Lion	89.0	0.38	0.48

### Analysis of convergence stability across vision tasks with varying complexity

To further validate adaptability of proposed optimizer enhancement strategies in complex vision scenarios, this section conducts experimental analysis on a higher-complexity vision task and a larger-scale vision dataset, respectively. Former is employed to examine convergence behavior under conditions of richer class structures and deeper model architectures, whereas latter is used to evaluate generalization capability and stability on larger-scale training samples. This task is based on natural images, with increased numbers of training and testing samples, thereby imposing higher requirements on convergence speed, overfitting control, and generalization capability of optimizers^26^.

Experimental model adopts vision transformer large architecture^27^,featuring parameter scales exceeding 300M and layer depth reaching 24 layers, serving as suitable platform to evaluate dynamic scheduling capabilities during complex model training processes. All optimizers uniformly employ learning rate of 1e-4, batch size of 512, maintaining experimental configurations consistent with mainstream literature to preclude additional bias stemming from model structure or task complexity. Experimental results are presented in Table 2. To examine performance of proposed methods on higher-complexity vision tasks, this study further conducts supplementary validation experiments on ImageNet-1K. Due to limitations in computational cost and training time, ResNet-50 is adopted as backbone model, and comparative evaluation is performed among Ranger21, Lion, Entropy-Cosine, Entropy-ReduceLROnPlateau, and MSCut, with results presented in Table 3.

**Table 2:** Performance comparison of different optimizers on more complex vision tasks.

Optimizer	ACC (%)	T-loss	V-loss
Ranger21	79.2	1.65	1.81
Ranger21-Entropy-Cosine	83.0	1.33	1.40
Ranger21-Entropy-ReduceLROnPlateau	82.6	1.35	1.43
EAGrad	82.0	1.38	1.48
Ranger21-MSCut	82.3	1.36	1.44
Lion	81.5	1.44	1.52

Notably, Lion optimizer yields a noticeable improvement over Ranger21 in this task, as reflected by clear reductions in both training and validation loss, further confirming its effectiveness in suppressing gradient fluctuations under large-scale settings. Nevertheless, optimization strategies incorporating entropy mechanisms deliver additional gains in training stability and generalization performance. Among them, Entropy-Cosine and Entropy-Plateau both achieve accuracy superior to that of Lion.

**Table 3:** Performance comparison of different optimizers on large-scale vision validation tasks.

Optimizer	ACC (%)	T-loss	V-loss
Ranger21	76.4	1.92	2.05
Ranger21-Entropy-Cosine	77.8	1.76	1.88
Ranger21-Entropy-ReduceLROnPlateau	77.5	1.79	1.91
Ranger21-MSCut	77.3	1.81	1.93
Lion	77.1	1.84	1.96

Results indicate that Lion exhibits more stable optimization capability than Ranger21 in complex vision tasks, while introduction of entropy mechanisms leads to further improvements across both accuracy and loss metrics. Among them, Entropy-Cosine demonstrates favorable overall performance across both categories of vision tasks, and MSCut maintains stable effectiveness in validation loss control, indicating that entropy-based scheduling and entropy-aware clipping exert positive effects on training of complex vision models. Overall, proposed methods show effectiveness across medium-scale and higher-complexity vision tasks, although current validation remains constrained by training budget, model configurations, and data scale.

### Analysis regarding optimization dynamics within natural language understanding tasks

To evaluate versatility and transferability regarding proposed optimizer enhancement strategies within natural language understanding tasks, this section selects performance of language models during sentiment recognition and sentence inference tasks as analytical subject. Compared with vision tasks, natural language processing models exhibit heightened sensitivity toward training dynamics; particularly regarding semantic transfer and cross-domain generalization, such models impose more stringent requirements upon optimizer robustness.

Experiments employ two mainstream Transformer architectures, RoBERTa-base and RoBERTa-large^28^, undergoing fine-tuning on SST-2 and MNLI from GLUE benchmark tasks^29,30^, respectively. Training duration is set to 5 epochs with batch size of 32 and initial learning rate of 2e-5, while scheduler adopts warmup coupled with linear decay structure to ensure configurations align with mainstream pre-training fine-tuning pipelines. Tables 4 and 5 summarize performance outcomes across SST-2 and MNLI for various methodologies, reporting accuracy, training loss, and validation loss.

**Table 4:** Performance comparison among diverse optimizers on RoBERTa-base (SST-2) task.

Optimizer	ACC (%)	T-loss	V-loss
Ranger21	92.3	0.21	0.31
Ranger21-Entropy-Cosine	93.6	0.17	0.26
Ranger21-Entropy-ReduceLROnPlateau	94.1	0.13	0.21
EAGrad	93.2	0.19	0.27
Ranger21-MSCut	93.4	0.18	0.26
Lion	93.1	0.19	0.28

**Table 5:** Performance comparison among diverse optimizers on RoBERTa-large (MNLI) task.

Optimizer	ACC (%)	T-loss	V-loss
Ranger21	85.0	0.51	0.64
Ranger21-Entropy-Cosine	86.5	0.41	0.52
Ranger21-Entropy-ReduceLROnPlateau	86.8	0.44	0.55
EAGrad	86.3	0.46	0.58
Ranger21-MSCut	86.6	0.45	0.57
Lion	86.2	0.47	0.59

Results indicate that Lion optimizer continues to demonstrate robust performance during RoBERTa fine-tuning tasks, surpassing traditional methodologies. Simultaneously, proposed entropy-modulated optimizers further enhance accuracy across both SST-2and MNLI tasks while effectively reducing training and validation loss, demonstrating that such methods possess favorable generalization capabilities within linguistic tasks as well.

### Computational overhead analysis

To evaluate practical overhead of proposed methods, this study measures runtime and GPU memory consumption of different optimizers. To reduce influence of model architecture on results, this part adopts ResNet-50 as backbone model, and relative overhead is computed with Ranger21 as reference baseline. Average training time per epoch, peak GPU memory usage, and additional overhead relative to Ranger21 are reported, with results presented in Table 6.

**Table 6:** Comparison of computational overhead across different optimizers.

Optimizer	Avg. time/epoch (s)	Peak GPU memory (GB)	Relative time overhead (%)	Relative memory overhead (%)
Ranger21	142	6.8	0.0	0.0
Ranger21-Entropy-Cosine	148	7.0	4.2	2.9
EAGrad	146	6.9	2.8	1.5
Ranger21-MSCut	153	7.2	7.7	5.9
Lion	138	6.6	-2.8	-2.9

Overall, entropy-enhanced methods introduce additional computational overhead relative to baseline optimizers, while magnitude of increase remains limited. Among them, Entropy-Cosine and EAGrad exhibit minor variations in runtime and memory usage, whereas MSCut incurs relatively higher additional cost, which is primarily associated with spectral feature estimation. Nevertheless, after adopting maximum singular value approximation and a low-frequency update strategy, overall overhead remains within an acceptable range.

**Fig. 3:**
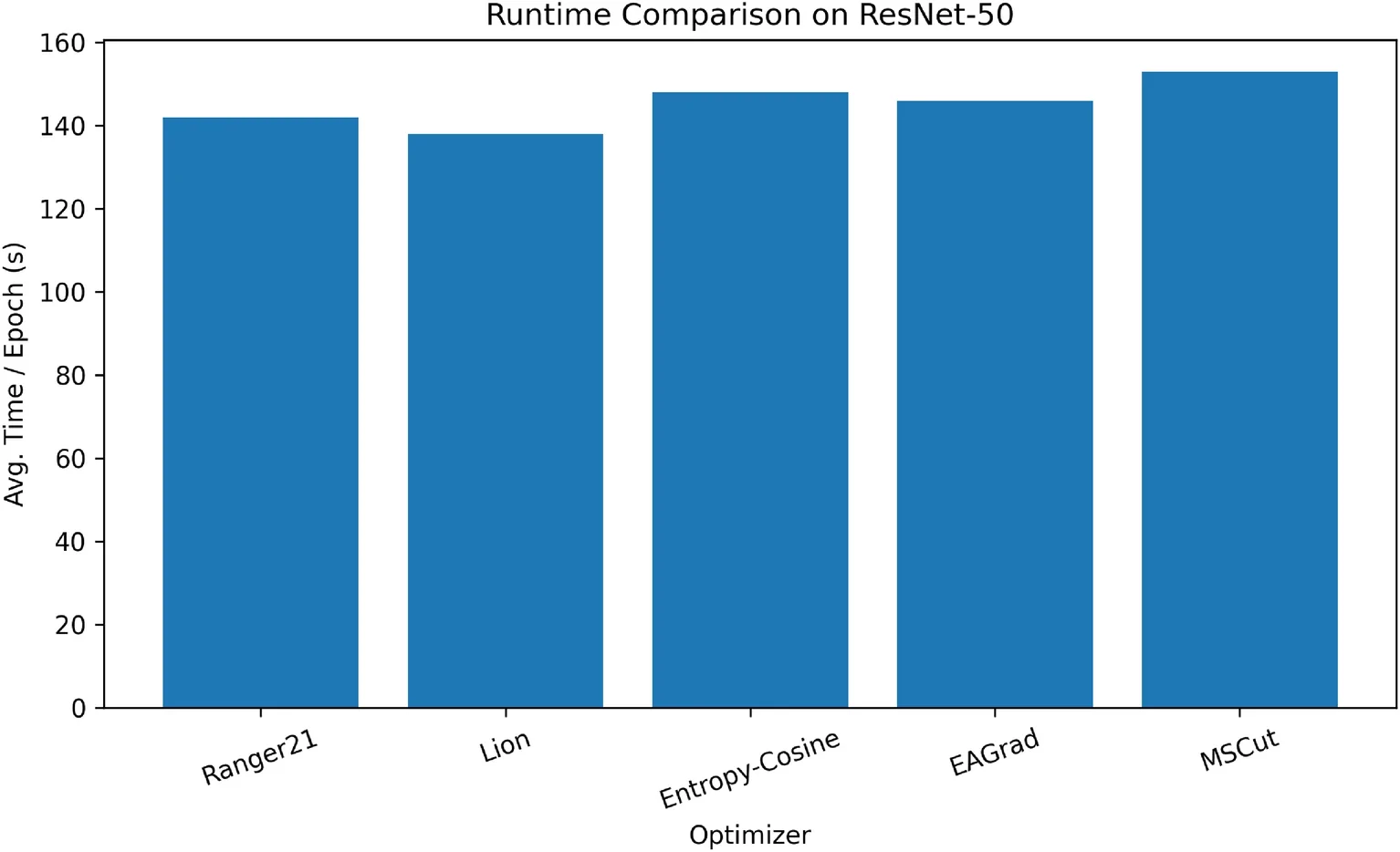
Comparison of average training time across different optimizers on ResNet-50.

**Fig. 4:**
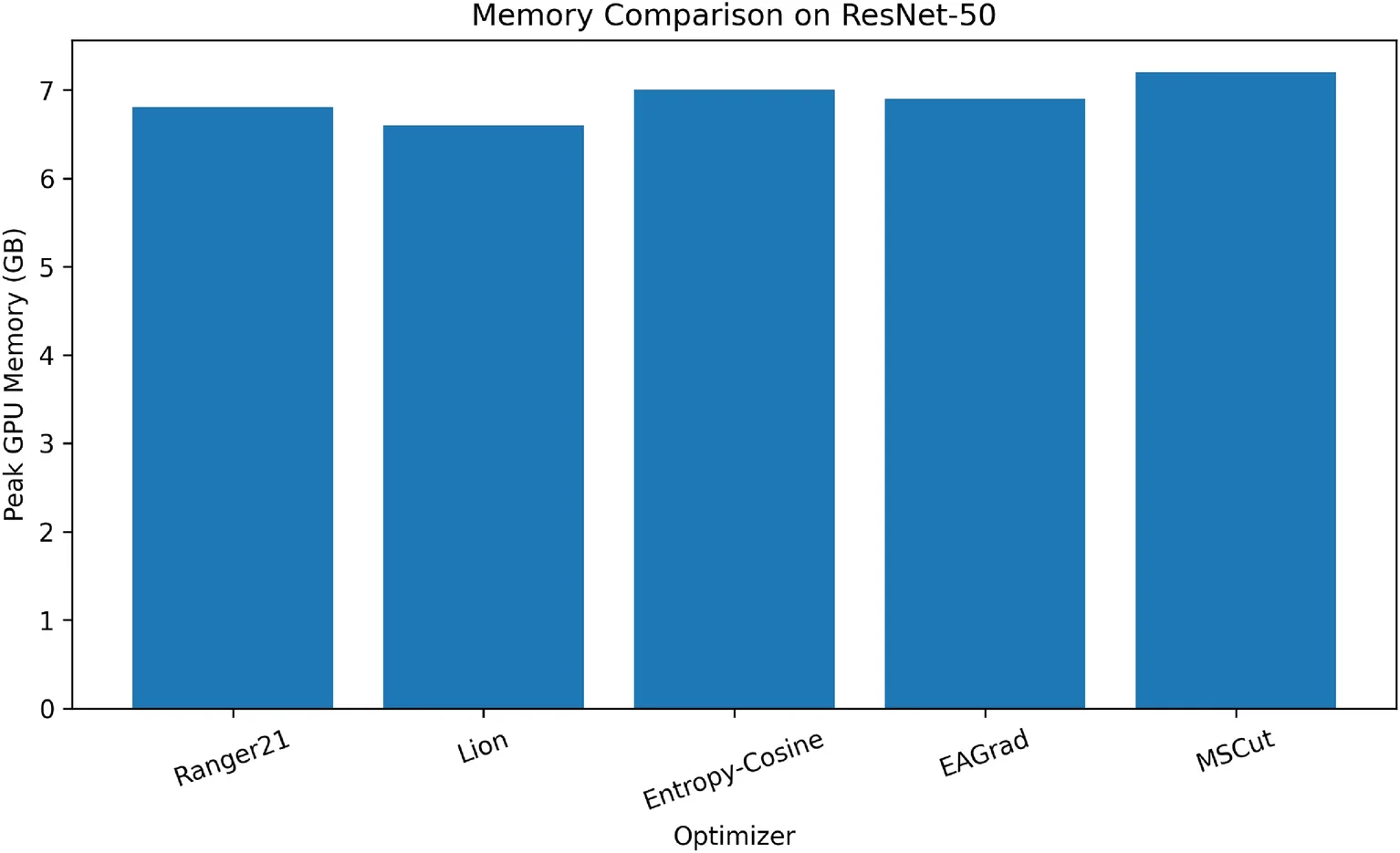
Comparison of peak GPU memory usage across different optimizers on ResNet-50.

Figures 3 and 4 present average training time and peak memory usage of different optimizers under ResNet-50, respectively. Entropy-cosine and EAGrad exhibit relatively moderate increases in computational overhead, whereas MSCut incurs slightly higher additional cost, while overall remaining within an acceptable range.

### Experimental summary and comprehensive analysis

Centering upon multiple vision and language tasks, present chapter systematically verifies effectiveness of proposed entropy-mechanism enhanced optimizers. Covering fundamental image classification, medium-scale vision tasks, and natural language understanding tasks, experiments incorporate contemporary lightweight optimizer Lion as additional baseline to conduct comparative analysis regarding performance of proposed methods across accuracy and training dynamics metrics.

Experimental results indicate that entropy-aware schedulers and gradient perturbation mechanisms significantly enhance training stability and validation accuracy across all tasks. Furthermore, judging from performance across diverse model scales and modalities, proposed methodologies possess robust versatility and transferability, facilitating dynamic regulation of parameter spaces within various architectures such as Transformer and ResNet to construct more flexible and perception-driven optimization paths. Notably, experiments on ImageNet-1K in this study are primarily conducted to validate effectiveness of proposed methods on larger-scale vision datasets, while related conclusions remain confined to current experimental settings and comparison conditions due to limitations in training budget and model configuration scope. Further analysis of training processes incorporating entropy metrics indicates that such mechanisms are capable of reflecting partial dynamic variations across training stages under current experimental settings and can provide auxiliary references for learning rate scheduling.

### Ablation study and stability analysis

To systematically evaluate functional roles and performance contributions of each submodule within overall optimizer architecture, module-level ablation experiments are conducted. An independent removal strategy is adopted, in which three key mechanisms in proposed optimizer, namely gradient perturbation rate modulation factor, information entropy scheduling mechanism, and entropy-aware clipping strategy, are sequentially removed. Under condition that backbone optimizer structure and model configuration remain unchanged, performance comparisons are carried out on three representative datasets, CIFAR-10, ImageNet, and SST-2, with results presented in Table 7.

**Table 7:** Independent contribution assessment regarding individual modules toward optimizer performance.

Experiment settings	CIFAR-10 *↓*%	ImageNet *↓*%	SST-2 *↓*%
w/o MSCut	− 0.7	− 0.9	− 0.8
w/o $H_t$	− 1.2	− 1.6	− 1.4
w/o $P_t$	− 1.0	− 1.3	− 1.2
w/o $P_t$ $H_t$	− 2.4	− 2.9	− 2.6

Results reveal that removal of information entropy mechanisms exerts most significant impact upon model performance, with average accuracy declines exceeding 1.2% across three datasets, indicating that such mechanisms play most critical role within dynamic regulation of schedulers. By contrast, perturbation rate modulation factors exhibit higher sensitivity across medium-to-large-scale datasets, where performance degradation resulting from removal generally surpasses observations recorded on CIFAR-10. Impact of MSCut module remains relatively modest, yet continues to provide certain marginal gains in specific tasks, particularly demonstrating unique advantages regarding stability control during training phases.

Furthermore, to evaluate performance of proposed methods under complex gradient environments, supplementary gradient noise perturbation experiments are conducted on CIFAR-10. Gaussian noise with varying intensities is added to gradients obtained during backpropagation, and performance variations of Ranger21, Lion, Entropy-Cosine, and EAGrad under noisy conditions are compared, with results presented in Table 8. As noise level increases, accuracy of all methods declines to some extent, whereas entropy-enhanced methods exhibit a relatively smoother degradation trend. Among them, Entropy-Cosine maintains comparatively stable performance across different noise conditions, and EAGrad, after incorporating additional gradient perturbation, also outperforms Ranger21. These observations indicate that gradient entropy and perturbation rate can, to a certain extent, reflect variations in gradient environments and provide auxiliary regulation for optimization processes.

**Table 8:** Accuracy comparison under different gradient noise levels.

Optimizer	Noise=0	Noise=0.01	Noise=0.05
Ranger21	88.3	87.5	85.9
Ranger21-Entropy-Cosine	90.1	89.6	88.5
EAGrad	88.8	88.2	87.1
Lion	89.0	88.4	87.0

Finally, to further evaluate statistical stability of experimental results, key methods are repeated using three different random seeds on two representative tasks, CIFAR-10 and SST-2, with results presented in Table 9. Performance variations across different random seeds remain relatively small for all methods, while entropy-enhanced methods exhibit comparatively smaller fluctuation ranges while maintaining high average performance.

**Table 9:** Comparison of performance stability across different random seeds.

Optimizer	CIFAR-10 ACC	SST-2 ACC
Ranger21	88.2 ± 0.18	92.2 ± 0.14
Ranger21-Entropy-Cosine	90.0 ± 0.12	93.7 ± 0.10
EAGrad	88.8 ± 0.15	93.2 ± 0.11
Lion	89.0 ± 0.16	93.1 ± 0.12

## Discussion

This study addresses optimizer performance enhancement from two fundamental perspectives: learning rate scheduling and parameter update strategy. By embedding information entropy mechanisms within CosineAnnealingLR and ReduceLROnPlateau, adaptability of these schedulers to phase-wise fluctuations during training is effectively enhanced. Particularly under non-stationary training processes, this approach reduces oscillations during training and improves convergence speed of models. Particularly in non-stationary training processes, this approach significantly reduces oscillations during training and enhances model convergence speed. EAGrad dynamically adjusts the learning rate through gradient perturbation rate and local information entropy, effectively compensating for the insufficient responsiveness of traditional optimizers during early training stages. MSCut employs entropy as a sensitivity estimation basis to guide adaptive adjustment of gradient clipping thresholds across layers and training phases. This design not only enhances the stability and generalization capability of the optimizer, but also advances the transition of optimizers toward information-aware regulators. Gradient noise experiments further indicate that proposed methods maintain a relatively smooth performance degradation trend in presence of additional gradient perturbations. This observation suggests that gradient entropy and perturbation rate can provide a certain degree of representation capability for training states at an empirical level. Within the context of this study, the introduction of information entropy is not merely a regulatory mechanism, but also a strategic embodiment of structurally intelligent optimization.

EAGrad enhances learning rate mechanisms by incorporating gradient perturbation rate and information entropy, thereby improving responsiveness to training dynamics and structural complexity while avoiding rapid annealing observed in traditional Adagrad during non-stationary phases. Compared with methods that rely on explicit inner-loop optimization or full curvature modeling, this approach incurs relatively limited additional computational cost under current implementation. Therefore, it demonstrates stronger engineering practicality. Chaudhari et al. proposed entropy-SGD by introducing an entropy regularization term to construct a generalized flat path on the loss surface, but its reliance on inner-loop optimization leads to high computational overhead, limiting applicability to large-scale tasks. Meanwhile, Ahmed et al. demonstrated that in policy optimization, entropy mechanisms help smooth loss curves and relax barriers between local optima, providing theoretical support for learning rate enhancement^31^. EAGrad integrates gradient perturbation rate and information entropy into update rules, enabling real-time responsiveness and structure-aware adaptation under lightweight computation, thus overcoming efficiency and adaptability bottlenecks of the above methods. On the other hand, in gradient clipping, AutoClip focuses on adaptively setting thresholds based on historical gradient norms but overlooks differences in model layer characteristics^32^. In contrast, MSCut determines clipping thresholds using gradient variance, information entropy, and singular values of parameter tensors, enabling hierarchical perception and adaptive clipping across different depths and training stages, balancing stability and generalization performance.

## Conclusion

Focusing on learning rate degradation and training instability in optimizers under complex tasks, this study proposes an enhanced framework integrating entropy-based learning rate schedulers and optimizer mechanisms. Proposed methods are overall superior to baseline optimizers across multiple datasets and evaluation metrics. Certain limitations remain within present research. Firstly, although proposed entropy-aware schedulers effectively enhance training stability and convergence speed, currently utilized scheduler types are restricted to CosineAnnealingLR and ReduceLROnPlateau. Future investigations could further explore more sophisticated entropy-mechanism scheduling strategies to improve adaptability toward varying training phases and task complexities. Secondly, experiments primarily focus upon a single baseline optimizer; integrating proposed techniques with other state-of-the-art optimizers could more extensively verify effectiveness.

Future work may expand across three dimensions: first, extending such mechanisms to multi-modal fusion tasks to construct task-aware scheduler frameworks; second, combining reinforcement learning or NAS (neural architecture search) to automatically synthesize diverse scheduling strategies, forming end-to-end learnable optimization systems; and third, introducing shared-independent entropy modeling mechanisms within multi-task learning to enhance optimizer generalization capabilities under cross-task collaborative training.

## Data Availability

The ImageNet-100dataset used in this study can be obtained via the following URL: https://www.modelscope.cn/datasets/tany0699/mini_imagenet100.
